# A 0.0014 mm^2^ 150 nW CMOS Temperature Sensor with Nonlinearity Characterization and Calibration for the −60 to +40 °C Measurement Range

**DOI:** 10.3390/s19081777

**Published:** 2019-04-13

**Authors:** Wendi Yang, Hanjun Jiang, Zhihua Wang

**Affiliations:** Institute of Microelectronics, Tsinghua University, Beijing 100084, China; yangwd13@mails.tsinghua.edu.cn (W.Y.); zhihua@tsinghua.edu.cn (Z.W.)

**Keywords:** CMOS temperature sensor, ultra-low power, nonlinear calibration, cold chain

## Abstract

This work presents a complementary metal–oxide–semiconductor (CMOS) ultra-low power temperature sensor chip for cold chain applications with temperatures down to −60 °C. The sensor chip is composed of a temperature-to-current converter to generate a current proportional to the absolute temperature (PTAT), a current controlled oscillator to convert the current to a frequency signal, and a counter as the frequency-to-digital converter. Unlike the conventional linear error calibration method, the nonlinear error of the PTAT current under the low temperature range is fully characterized based on the device model files provided by the foundry. Simulation has been performed, which clearly shows the nonlinear model is much more accurate than the linear model. A nonlinear error calibration method, which requires only two-point calibration, is then proposed. The temperature sensor chip has been designed and fabricated in a 0.13 μm CMOS process, with a total active die area of 0.0014 mm^2^. The sensor only draws a 140 nA current from a 1.1 V supply, with the key transistors working in the deep subthreshold region. Measurement results show that the proposed nonlinear calibration can decrease the measurement error from −0.9 to +1.1 °C for the measurement range of −60 to +40 °C, in comparison with the error of −1.8 to +5.3 °C using the conventional linear error calibration.

## 1. Introduction

Temperature monitoring is mandatory in cold chain applications for the production, storage, distribution, and transportation of perishable, but life-critical products, such as foods, blood products, and vaccines [1,2,3,4]. The temperature range for the food cold chain is commonly from −20 to +15 °C [1,2]. For blood products, such as fresh frozen plasma, the cold chain needs to maintain a low temperature under −25 °C. Temperature control for the vaccine cold chain is even more stringent [3], and some special vaccines, such as the anti-Ebola vaccine, may need a low temperature of −60 °C [4]. 

The fundamental requirement of temperature monitoring in cold chains is a high accuracy. Low power consumption is another key requirement, since in many cold chain applications, the temperature monitoring function is incorporated into the extremely power-constrained wireless telemetry circuit powered by a miniature battery or a radio frequency identification (RFID) tag based on wireless power transfer [5]. In addition, cold chain applications need temperature monitoring for every truck and even every small product package, and such applications are usually cost sensitive [5,6,7,8]. In general, there is a great need to investigate low-cost, low-power, wide-range, and high-accuracy temperature sensors for cold chain applications [9]. The target of this work is to develop a technology to design such a temperature sensor using the complementary metal–oxide–semiconductor (CMOS) technology.

There are many physical devices, such as a bipolar junction transistor (BJT) and CMOS transistor, that can be used to build monolithic temperature sensors, since these devices have temperature dependent properties that can indicate the environment temperature [10]. Temperature sensors based on the BJT have the advantages of a high accuracy and fast conversion speed, but BJT-based sensors usually consume high power and occupy large chip areas. For example, the BJT-based sensor in [11] occupies an area of 0.16 mm^2^ and consumes 6.9 μW power, which can barely be provided by an RFID tag. The BJT-based sensor requires 1.5 V or higher power supply to work at low temperatures, since the base-emitter voltage, V_BE_, rises to about 0.8 V at −55 °C [12]. Due to the high voltage supply requirement, relatively large power consumption, and large chip area, the BJT-based sensor is not competitive for low-power and low-cost cold chain applications. 

Monolithic temperature sensors based on the temperature dependence of the CMOS transistor model parameters have the advantages of a low power and small chip area [10], especially emerging time-to-digital converter based CMOS sensors [13,14,15,16,17]. For example, the time-to-digital converter was used to quantize the temperature dependent delay of an inverter-chain in [13], and the sensor only consumed 0.28 μW power with a chip area of 0.022 mm^2^. The ring oscillator based sensor in [15] has an even smaller power consumption of 0.2 μW with a chip area of 0.004 mm^2^. However, time-to-digital converter based sensors have a limited measurement range, and the lowest measurable temperature of the sensors in [13,14,15,16,17] is −40 °C, which cannot fulfill the cold chain application requirements. 

Conventional proportional-to-absolute-temperature (PTAT) current based CMOS temperature sensors [18,19] can extend the temperature measurement range to the lower end. For example, the PTAT current based sensor in [18] can measure the temperature down to −50 °C with 0.6 μW of power consumption and a chip area of 0.085 mm^2^.

Conventional PTAT based CMOS temperature sensors usually utilize one-point or two-point calibration to compensate for linear errors. However, our recent investigation reveals that it is not enough to just calibrate linear errors, and nonlinear errors will be relatively large when the measurement range of the PTAT based CMOS temperature sensor is extended down to the low temperature end, i.e., −60 °C, for cold chain applications.

In this paper, we present a CMOS ultra-low power temperature sensor for cold chain applications in temperatures down to −60 °C. The nonlinear error for the low temperature sensing will be characterized, and a two-point calibration method which can calibrate the nonlinear error will be presented. 

The remaining part of this paper is organized as follows. The circuit architecture of the presented CMOS temperature sensor is described in Section 2. The nonlinear error for low temperature measurement is characterized in Section 3. The calibration method is given in Section 4. The measurement results on the fabricated sensor chip are given in Section 5, followed by the conclusion in Section 6.

## 2. Temperature Sensor Circuit Architecture

**Figure 1:**
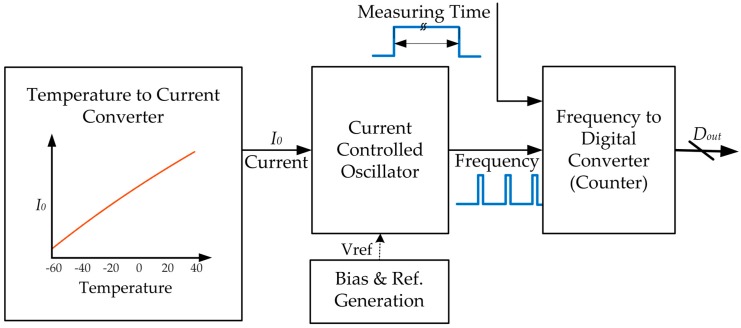
Block diagram of the temperature sensor.

The block diagram of the presented temperature sensor is shown in Figure 1. It includes three main functional blocks. The temperature-to-current converter generates a temperature-dependent current. Ideally, the output current is a PTAT current. A current controlled oscillator then converts the current to a frequency signal. The oscillation frequency is proportional to the PTAT current, and thus proportional to the absolute temperature. A counter serves as the frequency to digital converter, which digitizes the oscillator output frequency. In this work, the designed sensor is one function part of a system-on-a-chip (SoC), and it is powered by an on-chip 1.1 V low-dropout (LDO) regulator in the SoC or an external power supply. The design is optimized for the 1.1 V power supply.

**Figure 2:**
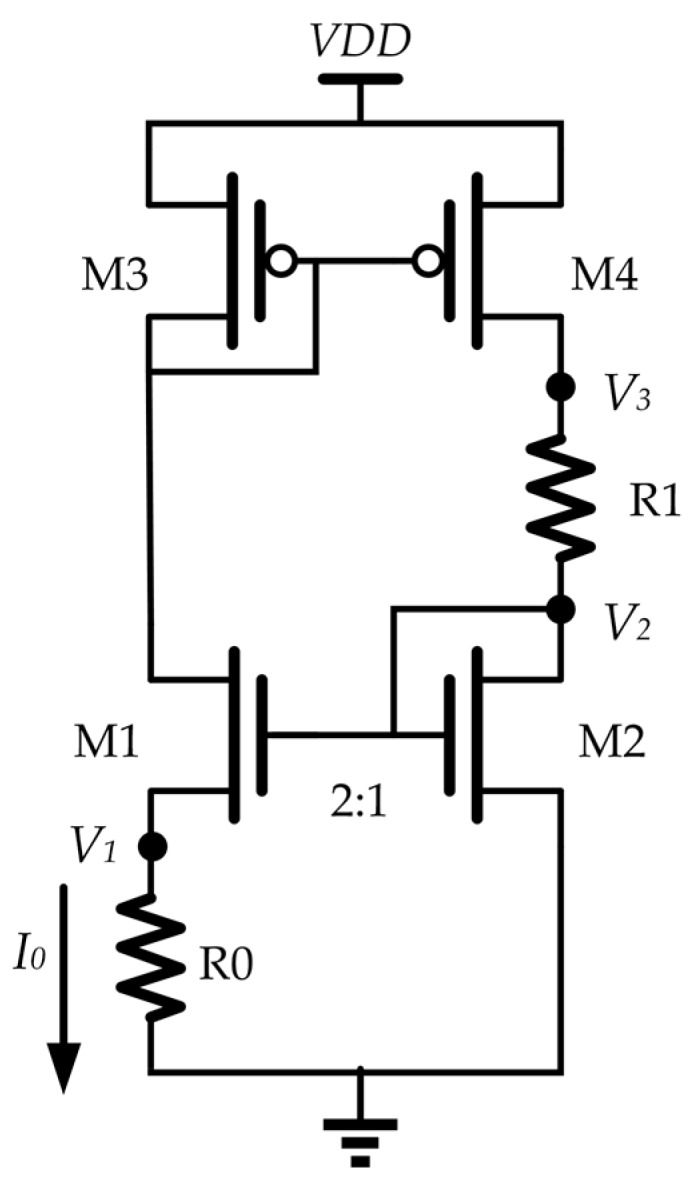
Circuit implementation of the temperature-to-current conversion.

The temperature-to-current converter is the key of this sensor. Figure 2 shows the circuit implementation of the temperature-to-current converter. M1 and M2 are working in the subthreshold region. The drain-source currents of M1 and M2 have the following relationship [20,21]:

(1) $$I_{M1}=\mu C_{ox}{\left(\frac{W}{L}\right)}_{1}V_{T}^{2}\exp\left(\frac{V_{2}-V_{1}-V_{th1}}{nV_{T}}\right)$$

(2) $$I_{M2}=\mu C_{ox}{\left(\frac{W}{L}\right)}_{2}V_{T}^{2}\exp\left(\frac{V_{2}-V_{th2}}{nV_{T}}\right)$$

where *μ* is the mobility, *C_ox_* is the oxide capacitance, *W*/*L* is the transistor aspect ratio, *V_th_*_1_ and *V_th_*_2_ are the threshold voltages of M1 and M2, respectively, and *n* is the subthreshold gate coupling coefficient. *V_T_* = *k T*/*q*, in which *k* is the Boltzmann constant, *T* is the absolute temperature, and *q* is the electron charge. As shown in Figure 2, *V*_1_ is the voltage across the poly resistor, R0, and *V*_2_ is the gate voltage of both M1 and M2. In this design, the aspect ratio of M1 is 2 times that of M2.

The current mirror formed by M3 and M4 is carefully matched. Ideally, M1 and M2 have the same current, which is denoted as *I*_0_:

(3) $$I_{0}=I_{M1}=I_{M2}$$

Substituting (1) and (2) into (3) leads to:

(4) $$V_{1}+\left(V_{th1}-V_{th2}\right)=n\cdot V_{T}\cdot ln2$$

If *V_th_*_1_ = *V_th_*_2_, then:

(5) $$V_{1}=n\cdot \frac{kT}{q}\cdot \ln2$$

If the resistance of R0 is constant, then the drain-source current of M1 is given by:

(6) $$I_{0}=\frac{V_{1}}{R}=\frac{n\cdot k\cdot \ln2}{qR_{0}}T$$

Ideally, *I*_0_ is a PTAT current [22,23,24], which is the basis of this type of temperature sensor [22]. A similar PTAT implementation in [24] showed the measured PTAT behavior.

## 3. Nonlinearity Characterization for a Wide Measurement Range

The validation of approximating *I*_0_ to a PTAT current relies on the assumptions that M1 and M2 have the same threshold voltages, and the resistance of R0 is not temperature dependent. These assumptions are approximately true for a modest temperature range [24]. However, when extending the measurement range to very low temperature, i.e., −60 °C, such an approximation actually introduces quite large nonlinearity. In this section, the nonlinearity of the temperature-to-current converter for a wide temperature range will be characterized.

Firstly, the transistor threshold voltage is temperature dependent. Based on the BSIM model [25,26,27,28], the threshold voltage of a MOS transistor is written as:

(7) $$V_{th}\left(T\right)=V_{th}\left(T_{0}\right)+\left(K_{T1}+\frac{K_{tl1}}{L_{eff}}\right)\left(\frac{T}{T_{0}}-1\right)+K_{T2}V_{bseff}\left(\frac{T}{T_{0}}-1\right)$$

where *K_T_*_1_, *K_tl_*_1_, and *K_T_*_2_ are process-dependent parameters, *L_eff_* is the effective channel length, and *V_bseff_* is the body-source voltage. Note that M1 and M2 have different source voltages, and the body-source voltage difference is just −*V_1_*. Taking the body effect into consideration [25,26,29,30], there exists a small difference between the threshold voltages of M1 and M2, which is given as:

(8) $$\Delta V_{th}\left(T\right)=K_{T2}\cdot \Delta V_{bseff}\left(\frac{T}{T_{0}}-1\right)=-V_{1}\frac{K_{T2}}{T_{0}}\left(T-T_{0}\right)$$

Substituting (8) into (4) leads to:

(9) $$V_{1}=\frac{n\cdot k\cdot ln2}{q}\cdot \frac{T}{1-\frac{K_{T2}}{T_{0}}\left(T-T_{0}\right)}=\frac{n\cdot k\cdot ln2}{q}\cdot \frac{T}{1+K_{T2}^{\prime }\left(T-T_{0}\right)}$$

in which *K_T_*_2_′ = −*K_T_*_2_/*T*_0_.

On the other hand, the temperature dependent resistance, *R*_0_ (*T*), at the temperature, *T*, is given by:

(10) $$R_{0}\left(T\right)=R_{0}\cdot \left[1+TC_{1}\cdot \left(T-T_{0}\right)+TC_{2}\cdot {\left(T-T_{0}\right)}^{2}\right]$$

where *R*_0_ is the resistance at the reference temperature, *T*_0_, and *TC*_1_ and *TC*_2_ are the temperature coefficients. Based on the foundry design kit (FDK), *TC*_1_ is on the order of 10^−5^, and *TC*_2_ is on the order of 10^−7^. The current, *I*_0_, through *R*_0_ is derived as:

(11) $$I_{0}=\frac{V_{1}}{R_{0}\left(T\right)}=\frac{n\cdot k\cdot ln2}{qR_{0}}\frac{T}{1+\left(K_{T2}^{\prime }+TC_{1}\right)\left(T-T_{0}\right)+\left(TC_{2}+K_{T2}^{\prime }TC_{1}\right){\left(T-T_{0}\right)}^{2}+K_{T2}^{\prime }TC_{2}{\left(T-T_{0}\right)}^{3}}$$

In the concerned temperature range of −60 to +40 °C, (*K_T_*_2_′ + *TC*_1_) · (*T* − *T*_0_) is much larger than (*TC*_2_ + *K_T_*_2_′ · *TC*_1_) · (*T* − *T*_0_)^2^ and *K_T_*_2_′ · *TC*_2_ · (*T* − *T*_0_)^3^. Equation (11) can be simplified by ignoring the high order terms in the denominator:

(12) $$I_{0}\approx \frac{n\cdot k\cdot ln2}{qR_{0}}\cdot \frac{T}{1+K_{TC}\left(T-T_{0}\right)}$$

in which *K_TC_* = *K_T_*_2_′ + *TC*_1_ is a process dependent constant.

The calculation based on the CMOS process technology file provided by the foundry indicates that *K_TC_* = 7.10 × 10^−5^ if *T*_0_ = 300 K. In the coefficient, *K_TC_*, the contribution of *K_T_*_2_′ due to the transistor threshold voltage temperature dependence and that of *TC*_1_ due to the resistor temperature dependence is about 70% and 30%, respectively.

It can be concluded that with the temperature dependence of the transistor threshold voltage and the resistance temperature dependence, the drain-source current of M1 cannot simply be treated as a PTAT current. Equation (12) indicates that the current-temperature curve has a hyperbolic shape. Though the FDK available only provides device models down to −40 °C, Equation (12) works for much lower temperatures since the BSIM model is valid for quite a wide temperature range [25].

Equation (12) also indicates that if the measurement range is small, then the measured temperature is close to the reference temperature, *T*_0_; *K_TC_* · (*T* − *T*_0_) is small; and the denominator, 1 + *K_TC_* · (*T* − *T*_0_), degenerates to 1. In that case, Equation (12) degenerates to a linear relationship between *I*_0_ and *T*, resulting in a PTAT current as expected. However, for the large measurement range targeted in this work, *K_TC_* · (*T* − *T_0_*) is not negligible, and the linear approximation will introduce quite large error, which requires calibration.

**Figure 3:**
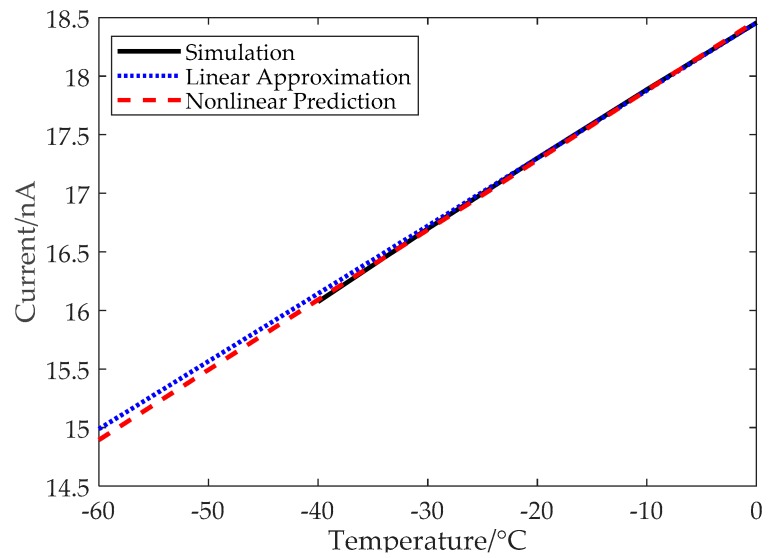
The simulated *I*_0_ vs. the temperature, the linear approximation of *I*_0_, and the hyperbolic prediction from (12).

Figure 3 shows the simulated current, *I*_0_, for the temperature range of −40 °C to 0 °C (the lower simulation boundary is limited by the FDK), in contrast to the conventional linear approximation and the hyperbolic prediction using (12). The black line is the simulated current, while the blue dashed line is a straight line by connecting two points (−20 °C and 0 °C) on the black line, and the red dashed line is the hyperbolic fitting of these two points. The difference between the simulated current and the straight line reaches 0.07 nA for the temperature of −40 °C, which means that the measurement error will be about 1.2 °C for the −40 °C point with conventional linear approximation. In contrast, the difference between the simulated current and the nonlinear prediction is only 0.02 nA at −40 °C, which corresponds to only a 0.34 °C measurement error. Obviously, the simulated current, *I*_0_, is closer to the hyperbolic line as predicted by (12) rather than the straight line. It can also be roughly calculated from Figure 3 that the measurement error using conventional linear approximation will reach 2.8 °C when the temperature is down to −60 °C.

## 4. Digitization and Calibration

**Figure 4:**
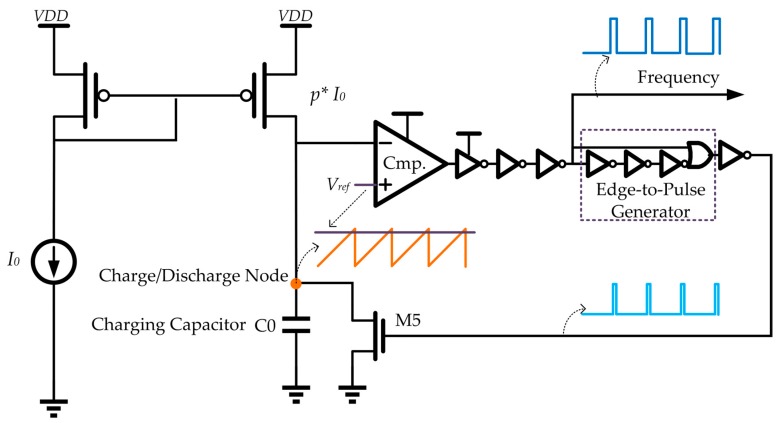
Circuit implementation of the current controlled oscillator.

The current controlled oscillator and the frequency-to-digital converter (counter) in Figure 1 are used to digitize the temperature dependent current, *I*_0_. As shown in Figure 4, the current controlled oscillator is implemented as a relaxation oscillator, taking advantage of the small area and low power consumption [31]. In the oscillator, the current, *I*_0_, is duplicated and amplified by *p* times to serve as the charging current. To ensure a linear relationship between the oscillation frequency and the charging current, *p* · *I*_0_, all the unwanted delays in the oscillator feedback loop are carefully optimized and reduced. An edge-to-pulse generator is inserted in the loop to shortly turn on the switch transistor, M5, and reset the sawtooth waveform at the charge/discharge node periodically.

Ideally, the oscillator output clock cycle period, *τ*, is calculated as:

(13) $$\tau =\frac{C_{0}\cdot V_{ref}}{p\cdot I_{0}}$$

where *C*_0_ is the timing capacitor at the charge/discharge node, *V_ref_* defines the sawtooth waveform magnitude, and *p* is the ratio of the current mirror. Considering the extra delay in the feedback loop, which is independent of the charging current, such as the delay caused by the comparators, the logic gates, and the discharging switch, M5, an offset component denoted as *τ_os_* should be added to the clock cycle period, τ:

(14) $$\tau =\frac{C_{0}\cdot V_{ref}}{p\cdot I_{0}}+\tau_{os}$$

In this work, the nominal value of the charging time, $\frac{C_{0}\cdot V_{ref}}{p\cdot I_{0}}$, is about 0.2 ms. Simulation shows that the extra delay contributed by the comparator, the logic gate, and the discharging switch is about 3.5 μs, 30 ns, and 3.5 ns, respectively. In general, *τ_os_* is much smaller than $\frac{C_{0}\cdot V_{ref}}{p\cdot I_{0}}$, and it follows that $\frac{\tau_{os}}{\frac{C_{0}\cdot V_{ref}}{p\cdot I_{0}}}\ll 1$. To make the following derivation simple, Equation (14) is re-arranged and approximated as:

(15) $$\tau =\frac{C_{0}\cdot V_{ref}}{p\cdot I_{0}}\left(1+\frac{\tau_{os}}{\frac{C_{0}\cdot V_{ref}}{p\cdot I_{0}}}\right)=\frac{C_{0}\cdot V_{ref}\cdot \left(1-{\left(\frac{\tau_{os}}{\frac{C_{0}\cdot V_{ref}}{p\cdot I_{0}}}\right)}^{2}\right)}{p\cdot I_{0}\cdot \left(1-\frac{\tau_{os}}{\frac{C_{0}\cdot V_{ref}}{p\cdot I_{0}}}\right)}\approx \frac{C_{0}\cdot V_{ref}}{p\cdot I_{0}\cdot \left(1-\frac{\tau_{os}}{\frac{C_{0}\cdot V_{ref}}{p\cdot I_{0}}}\right)}$$

Let $I_{\mathrm{OS}}=p\cdot I_{0}\cdot \frac{\tau_{os}}{\frac{C_{0}\cdot V_{ref}}{p\cdot I_{0}}}$, and then Equation (15) is simplified to:

(16) $$\tau \approx \frac{C_{0}\cdot V_{ref}}{p\cdot I_{0}-I_{\mathrm{OS}}}$$

In this design, the oscillator output clock is divided by *m* times, and then the number of oscillation cycles within a given measurement time, *τ_cnt_*, is counted by the frequency-to-digital converter (counter) in Figure 1. The length of $\tau_{cnt}$ is controlled by a reference clock. The nominal value of $\tau_{cnt}$ is 1000 ms, and *m* = 4. The counter output number, *D_out_*, is given as:

(17) $$D_{out}=\frac{\tau_{cnt}}{m\cdot \tau }$$

Combining Equations (16) and (17) yields:

(18) $$D_{out}=\frac{\tau_{cnt}\cdot p}{m\cdot C_{0}\cdot V_{ref}}\cdot I_{0}-\frac{\tau_{cnt}I_{\mathrm{OS}}}{m\cdot C_{0}\cdot V_{ref}}$$

Substituting Equation (12) into Equation (18) gives:

(19) $$D_{out}=\frac{pnk\tau_{cnt}ln2}{mqR_{0}C_{0}V_{ref}}\cdot \frac{T}{1+K_{TC}\left(T-T_{0}\right)}-\frac{\tau_{cnt}I_{\mathrm{OS}}}{mC_{0}V_{ref}}$$

It is not necessary to know the value of each individual parameters in Equation (19). Let:

(20) $$k=\frac{pnk\tau_{cnt}ln2}{mqR_{0}C_{0}V_{ref}}$$

(21) $$b=-\frac{\tau_{cnt}I_{\mathrm{OS}}}{mC_{0}V_{ref}}$$

Additionally, Equation (19) can be simplified as:

(22) $$D_{out}=k\cdot \frac{T}{1+K_{TC}\left(T-T_{0}\right)}+b$$

*T*′ is defined as:

(23) $$T^{\prime }=\frac{T}{1+K_{TC}\left(T-T_{0}\right)}$$

Then, Equation (22) can be written as:

(24) $$D_{out}=k\cdot T^{\prime }+b$$

It is anticipated that the values of *k* and *b* in Equation (24) vary randomly chip by chip with the random process variation, and other random effects, such as the reference clock period error. On the other hand, it can be seen from Section 3 that the value of the coefficient, $K_{TC}$, in Equation (23) can be viewed as deterministic for all the chips fabricated using a given process.

To find the actual temperature value, *T*, from the counter reading, *D_out_*, the following calibration procedure is applied to compensate for both the random linear errors in *k* and *b*, and the deterministic nonlinear error as analyzed in Section 3. A two-point calibration is used to calibrate the linear errors. That is to say, each temperature sensor chip needs to be measured at two exactly-known temperature points, *T*_1_ and *T*_2_.

Step 1. For the specific chip, under the first given temperature settling (20 °C in this work), use a high performance sensor to measure the temperature, T_1_, and record the counter reading, D_out1_, within a given measurement time;

Step 2. Under the second given temperature setting (−30 °C in this work), use a high performance sensor to measure the temperature, T_2_, and record the counter reading, D_out2_;

Step 3. Use Equation (23) to calculate $T_{1}^{\prime }=\frac{T_{1}}{1+K_{\mathrm{TC}}\left(T_{1}-T_{0}\right)}$ and $T_{2}^{\prime }=\frac{T_{2}}{1+K_{\mathrm{TC}}\left(T_{2}-T_{0}\right)}$. Note that K_TC_ = 7.10 × 10^−5^ with T_0_ = 300 K;

Step 4. Use the linear Equation (24) to calculate the values of k and b with (T_1_′, D_out1_) and (T_2_^′^, D_out2_) from Step 1 to 3;

Step 5. For each output value, D_out_, given by this sensor, use Equation (22) to calculate the actual temperature, T, with the obtained values of *k* and *b* from Step 4, and the values of *K_TC_* and *T*_0_ from Section 3.

The proposed temperature sensor has been designed as a part of a wireless sensing SoC, and the calculation in Steps 3 to 5 can be implemented by programming the embedded microcontroller (MCU) in the SoC. For those application scenarios without the MCU, the calibration function can be easily implemented using a small digital logic circuit.

## 5. Measurement Results

**Figure 5:**
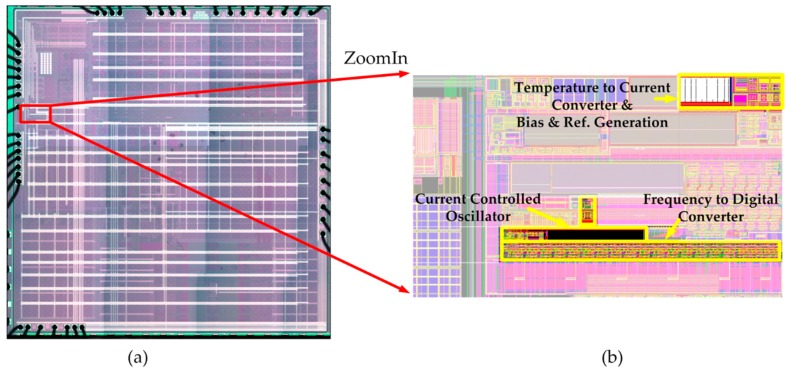
The SoC which contains the presented temperature sensor: (**a**) micrograph of the SoC; (**b**) layout of the presented temperature sensor.

The proposed temperature sensor chip was designed and fabricated as a function part of a SoC in a 0.13 μm CMOS process, and the die microphotograph of the SoC is shown in Figure 5a. Note that the temperature to current converter and the bias/reference generation circuit are shared by this temperature sensor and some of the other function parts in the SoC, and they are located apart from the current controlled oscillator and the frequency-to-digital converter, as shown in the sensor circuit layout given in Figure 5b. The total active area of the reported temperature sensor, including all the function blocks shown in Figure 1, is 0.0014 mm^2^. 

**Figure 6:**
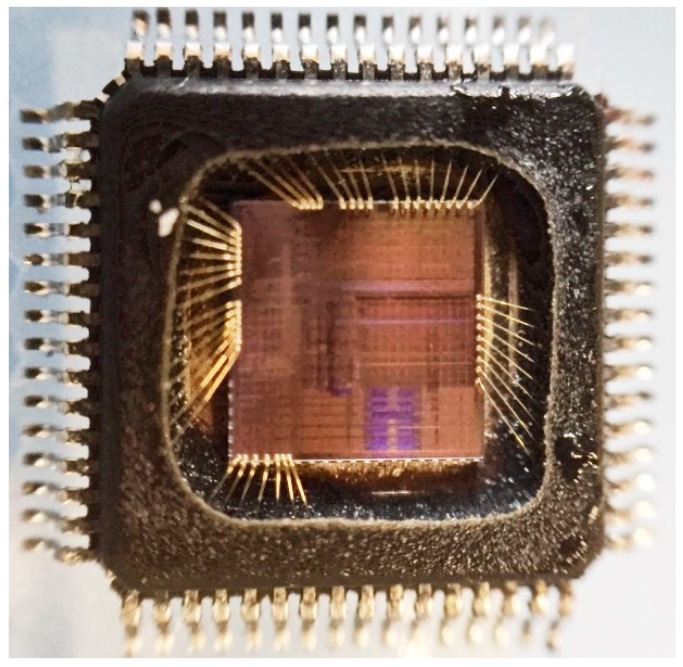
The SoC, which contains the presented sensor in a QFP-64 package (decapped).

For the test purpose, the SoC was packaged in a 64-pin quad flat package (QFP-64). A photo of the decapped QFP-64 package with the SoC die in it is given in Figure 6.

**Figure 7:**
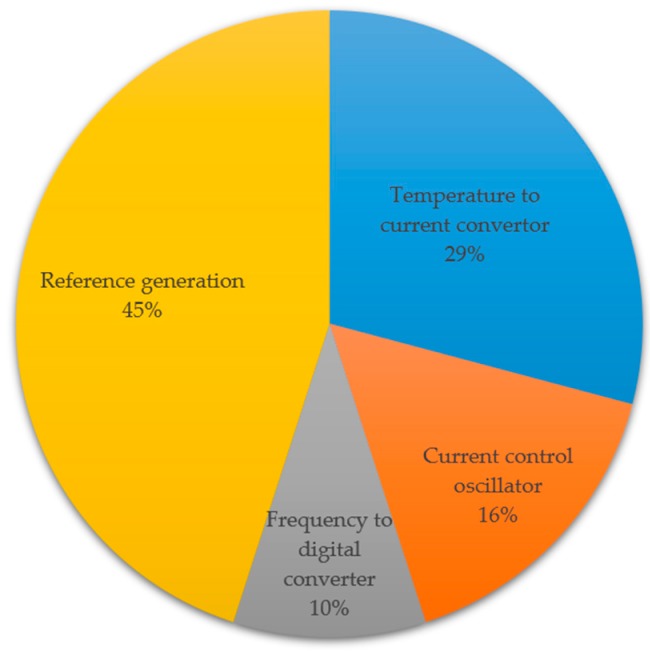
Power consumption breakdown of the presented temperature sensor chip (simulated).

The measured typical power consumption of the presented sensor is 0.15 μW (including all the function blocks shown in Figure 1), with a 1.1 V power supply at room temperature. The simulated power consumption breakdown is shown in Figure 7.

Four chips randomly selected from the same lot were measured to validate the presented nonlinearity characterization and calibration. The sampling rate in the measurement is 1 sample per second, with an external reference clock of 1 Hz generated by a crystal oscillator with less than 25 ppm temperature drift over the measured range of −60 °C to 40 °C. Actually, the reference clock frequency does not need to be quite this precise, since this frequency error will be cancelled out during the calibration.

**Figure 8:**
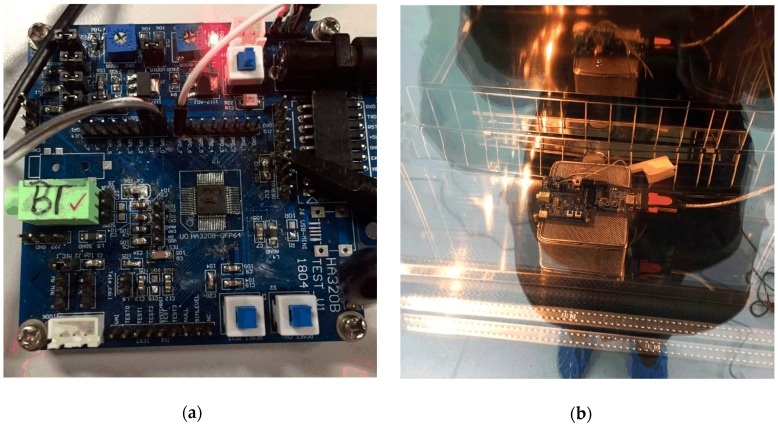
Experiment setup: (**a**) the PCB used to the test the sensor in the SoC in a QFP64 package; (**b**) the measurement PCB in the chamber.

The measurement was carried out by placing the temperature sensor printed circuit board (PCB) board inside a temperature and humidity chamber (model ASR-0220 manufactured by ESPEC). A T2000 handheld thermometer manufactured by Xiatech Electronics with ±0.1 °C inaccuracy was used to measure and set the chamber temperature. Figure 8 shows the PCB used to test the sensor, and the measurement environment (the chamber).

**Figure 9:**
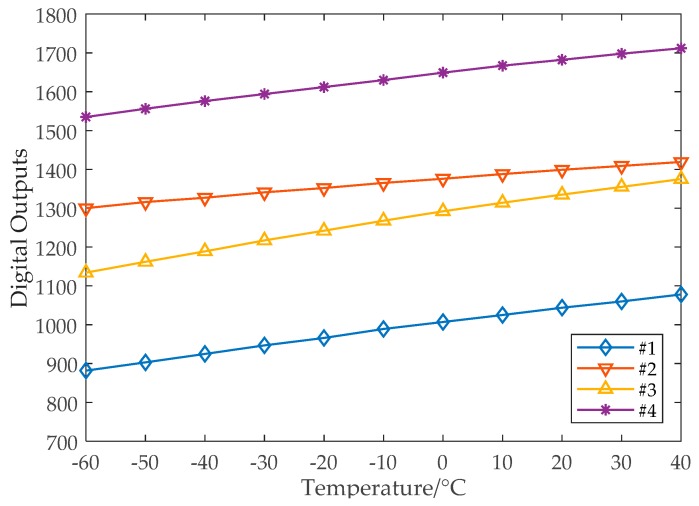
Measured temperature sensor outputs without calibration.

The measured temperature sensor outputs for the temperature range of −60 to 40 °C before the cycle-number-to-temperature conversion and calibration are shown in Figure 9. For the 100 °C range, the sensor digital output, $D_{out}$, has a maximum difference of about 200, which indicates a measurement resolution of 0.5 °C. Clearly shown in Figure 9, there exists random linear errors (the slope error and the offset error). Though not quite visible, further calculation shows that there also exists hyperbolic nonlinear error as predicted in Section 3.

All the measured chips were then calibrated with the data acquired at the temperature points of *T*_1_ = 20 °C and *T*_2_ = −30 °C. After obtaining the calibration parameters (*k* and *b*), the temperature range of −60 °C to 40 °C with a step of 10 °C was measured for each chip.

**Figure 10:**
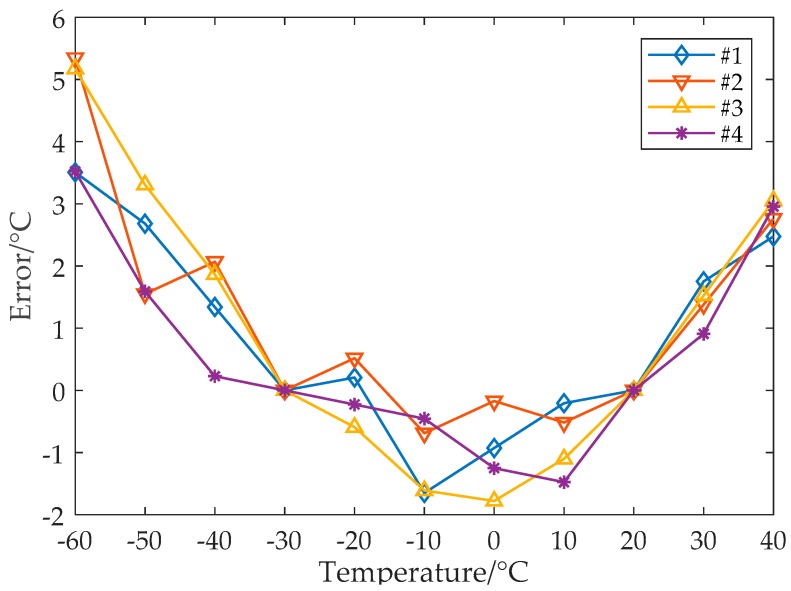
Temperature measurement error for the range of −60 to +40 °C with the conventional linear calibration.

Figure 10 shows the temperature measurement error after the conventional linear error calibration. Since the nonlinear error is not taken care of, the maximum measurement error reaches 5.3 °C (the full range error is −1.8 °C to +5.3 °C).

**Figure 11:**
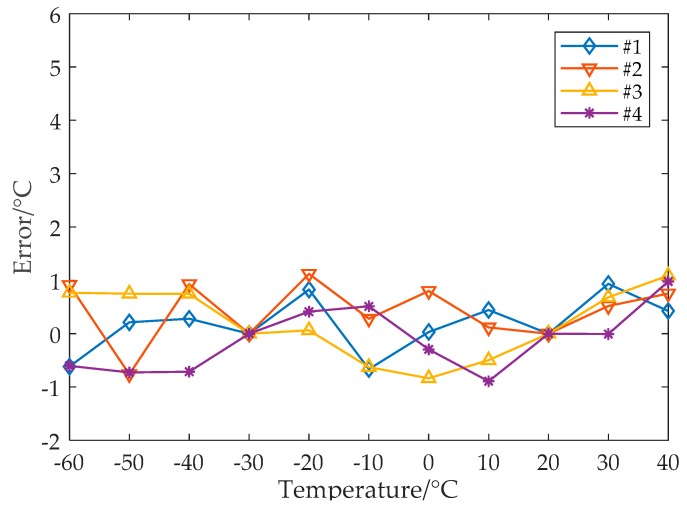
Temperature measurement error for the range of −60 to +40 °C with the proposed nonlinear calibration.

Figure 11 shows the temperature measurement error after the proposed nonlinear error calibration using the steps given in Section 4. The maximum measurement error decreases to only 1.1 °C (the full range error is −0.9 °C to +1.1 °C).

Note that the sensor design was optimized for the 1.1 V power supply. The measurement results shows that when the power supply deviates from the nominal 1.1 V by ±0.1 V, the oscillator output frequency may vary by ±0.4%, which corresponds to a temperature measurement error of about 1.2 °C. The presented sensor accuracy is sensitive to the power supply variation, and it needs to be used with the fixed power supply of 1.1 V for the best performance.

The performance of the presented temperature sensor chip is summarized and compared to other state-of-the-art designs in Table 1. Compared to other designs, the presented chip shows the lowest measurement temperature with the smallest chip area and the lowest power consumption, by using the proposed nonlinear error calibration. However, the power consumption reduction is actually at the cost of a narrowed measurement range at the high temperature end, which is clearly shown in Table 1. Note that the charging PTAT current was set quite small (~10 nA) to reduce the chip power consumption. On the other hand, the leakage current of the discharging switch transistor M5 approaches the charging current at high temperature. Consequently, the presented sensor design will fail at high temperatures.

## 6. Conclusions

In this paper, a CMOS temperature sensor was presented for temperature monitoring down to −60 °C in cold chain applications. The nonlinear error in the conventional PTAT current based sensor circuit was characterized for the first time, and a two-point calibration method was proposed to compensate for the nonlinear error in addition to the traditional linear error calibration. With the proposed nonlinear calibration, the measurement error decreased to −0.9 to +1.1 °C for the temperature range of −60 to +40 °C. The temperature sensor chip occupied a die area of 0.0014 mm^2^, and the typical power consumption was only 0.15 μW from a 1.1 V power supply, which outperforms similar designs in the literature.

## Figures and Tables

**Table 1:** Performance summary.

Sensor	[13]	[14]	[15]	[16]	[17]	[18]	[19]	This Work
CMOS technology (nm)	65	180	180	65	350	180	160	130
Area (mm^2^)	0.022	0.089	0.05	0.004	0.09	0.475	0.085	0.0014
Inaccuracy (°C)	2.6	2.0	4.6	1.8	1.2	1.6	0.8	2.0
Temp. range (°C)	0~100	−20~80	0~100	0~100	−40~60	−50~150	−40~125	−60~40
Conversion rate (sps)	40	1.3	10	45 k	5	100	166.7	1.0
Power consumption (μW)	0.28	0.8	0.2	154	1.5	69	0.6	0.15 *
Supply voltage (V)	0.4	1.8	1.8	1	3.0	1.5	0.85	1.1
Resolution (mK)	250	90	300	300	90	130	63	500
Number of samples	8	10	5	7	16	8	16	4
Trimming points	2	2	2	2	2	1	1	2

* Including the power consumption of all the function blocks shown in Figure 1, but not including that of the digital calibration function.
